# Altered structural and transporter-related gene expression patterns in the placenta play a role in fetal demise during Porcine reproductive and respiratory syndrome virus infection

**DOI:** 10.1186/s12864-025-11397-0

**Published:** 2025-03-21

**Authors:** Angelica Van Goor, Alex Pasternak, Kristen E. Walker, Shannon Chick, John C. S. Harding, Joan K. Lunney

**Affiliations:** 1 Animal Parasitic Diseases Laboratory, Beltsville Agricultural Research Center, ARS, USDA, Beltsville, MD USA; 2 Department of Animal Sciences, Purdue University, West Lafayette, IN USA; 3 Department of Large Animal Clinical Sciences, Western College of Veterinary Medicine, University of Saskatchewan, Saskatoon, SK Canada; 4 Present Address: Division of Animal Systems, Institute of Food Production and Sustainability, NIFA, USDA, Kansas City, MO USA

**Keywords:** Angiogenesis, Arachidonic acid, Disease resistance, Fetal pig, Gene expression, Placenta, Porcine reproductive and respiratory syndrome, Solute carrier proteins, Structural pathways, Intercellular junctions

## Abstract

**Background:**

Porcine reproductive and respiratory syndrome virus (PRRSV) can be transmitted across the maternal-fetal-interface from an infected gilt to her fetuses. Although fetal infection status and disease outcomes vary, the mechanisms are not completely understood. The objective was to assess targeted placental structural and transporter-related gene expression patterns. At day 85 of gestation pregnant pigs were challenged with PRRSV, and at 12 days post maternal infection sows and fetuses were sacrificed, and the placental tissue was collected. Grouping of fetuses was by preservation status and PRRS viral load (VL): control (CTRL, *n* = 14), viable and low VL fetus (VIA_LVF, *n* = 15), viable and high VL fetus (VIA_HVF, *n* = 21), meconium mild and low VL fetus (MECm_LVF, *n* = 14), meconium mild and high VL fetus (MECm_HVF, *n* = 14), and meconium severe and high VL fetus (MECs_HVF, *n* = 13). NanoString was used to evaluate the expression of 86 genes: actin cytoskeleton signaling, arachidonic acid pathway, integrin signaling, intercellular junctions, transporters, and VEGF signaling. Statistical analyses were performed using Limma with *P* ≤ 0.05 considered significant.

**Results:**

We identified 1, 7, 0, 29, and 39 differentially expressed genes in VIA_LVF, VIA_HVF, MECm_LVF, MECm_HVF, and MECs_HVF, respectively, contrasted to CTRL. Placental transporter genes were significantly impacted (i.e., downregulation of *SLC1A3*, *SLC1A5*, *SLC2A1*,* SLC2A3*,* SLC2A5*,* SLC2A10*,* SLC2A12*,* SLC7A4*,* SLC16A5*, *SLC16A10*, and *SLC27A6;* and upregulation of *SLC2A2*,* SLC16A3*, and *SLC27A4)*, compared to CTRL. Actin cytoskeleton signaling (*ARHGEF6* and *ARHGEF7)*, arachidonic acid (*PTGES3* and *PTGIS*), integrin signaling (*FN1* and *ITGB6*), intercellular junctions (*CDH3* and *CDH11*), and VEGF signaling (*MAPK3* and *HPSE*) gene groupings were significantly impacted, compared to CTRL.

**Conclusion:**

Data reported here indicate that fetal PRRSV infection levels rather than fetal demise is necessary for transcriptional dysregulation of the fetal placenta, with a tendency towards more downregulation in the target gene sets among susceptible fetuses. These results generally support that in susceptible fetuses there is altered solute transportation, placental structural integrity, and reduced angiogenesis. The data described here is associated with fetal PRRS resistance/resilience and susceptibility.

**Supplementary Information:**

The online version contains supplementary material available at 10.1186/s12864-025-11397-0.

## Background

Characterized by reproductive failure in pregnant gilts and respiratory illness in growing pigs, porcine reproductive and respiratory syndrome (PRRS) is a viral disease that results in significant economic losses, last estimated at $664 million dollars annually in the U.S. alone [1, 2]. The PRRS virus (PRRSV) is highly infectious, extremely mutable, pathologically variable, and persistent. Thus far, vaccination and biosecurity have been somewhat effective in reducing losses from PRRS. However, additional opportunities exist, especially in pregnant pigs, where incorporation of favorable genetics and an improved understanding of host-pathogen interactions are needed to improve outcomes on the farm.

PRRSV most efficiently infects the fetus during the late gestation (last 1/3), which can result in fetal mortality, premature farrowing, stillbirth, and postnatal failure to thrive [3–5]. Once virus infects the fetus, it can be found in most, if not all, lymphoid and parenchymal fetal tissues including blood, lymph nodes, and thymus [6]. Previously, PRRSV was reported to be transmitted from dam to fetal placenta within 2 days post maternal infection (DPMI), was found in fetal thymus by 8 DPMI, and 73% (36 of 49) of fetuses had detectable virus in their serum by 12 DPMI [7]. Fetuses at 12 DPMI can be differentially impacted by PRRSV infection as assessed by viral loads (VL) and fetal preservation, demise, or compromise (meconium staining: MEC). These fetuses were more likely to succumb to the infection if they had high VL and their neighboring fetuses were highly infected [8].

Debate exists related to the cause of fetal demise; specifically if viral effects within the fetus, the maternal fetal interface (MFI: maternal endometrium and/or fetal placenta), or both are responsible [9]. Previous work from our group supported the concept that fetal demise in response to PRRSV type 2 (PRRSV2) infection are determined more significantly by the placental response, rather than the thymic response which is initiated only after fetal infection [10]. The placenta is a dynamic organ that is responsive to fetal needs and the thymus is the presumed sight of replication within PRRSV infected fetuses [4]. Recent work supports the association of fetal PRRS VL in non-lymphoid tissues with fetal outcome; VL in the heart, brain, lung and skeletal muscle were significantly higher in more susceptible fetuses, and fetal infection causes rather significant physiological disruption in critical organ and endocrine systems [6].

The pig has a non-invasive, epitheliochorial placenta with a diffuse distribution of chorionic villi and non-invasive attachment of fetal and maternal tissues. In order to meet the needs of the developing conceptus, the porcine placenta using a combination of mechanisms including passive diffusion for soluble gasses, facilitated diffusion for simple biomolecules, active transport for charged ions, and spatially specific endo/pinocytosis for complex macromolecules. At the same time the placenta creates a structural barrier, largely isolating the conceptus from the maternal immune system and the majority of microorganisms. In the case of PRRSV, the mechanism of vertical transmission across the placenta is not completely understood. However, previous research has shown that PRRSV is detected in both the pig fetal placenta and maternal endometrium and causes apoptosis of infected and surrounding cells [11]. However, it is uncertain whether apoptosis within the MFI is a significant factor in viral transmission or a biproduct of the fetal response to infection. Even though the mechanism of viral transmission from mother to fetus is not completely understood, there is an association with fetal infection and the number of sialoadhesion CD169 and CD163 positive macrophages (i.e., PRRSV permissible cells) present at the MFI [9, 12]. Thus, critical questions remain with regards to the mechanisms of transplacental PRRSV infection and the role of tissues at the MFI (e.g., placenta) in fetal resilience/tolerance following infection.

Studies using gene expression have found common themes during fetal PRRSV infection, including changes to innate and adaptive immunity, interferon signaling, endocrine dysregulation, hypoxia, and apoptosis [13–16]. This present study tested placental genes in six structural and functional gene groupings to better understand their impact in fetal demise. The actin cytoskeleton and the associated signaling transduction pathways are involved in regulation of cellular shape, movement, and pathogen cell invasion among other functions [17]. Arachidonic acid is an essential fatty acid, a major component of cell membranes and a substrate for the synthesis of eicosanoids including prostaglandins [18]. Integrins are transmembrane proteins that mediate cellular adhesion [19], and play a vital role in placental attachment. Tight junctions and other intercellular adhesion molecules form the complex pericellular structure that controls permeability [20]. Solute carriers (SLCs) function in the facilitated transport of glucose, amino acids, and fatty acids as they are among some of the most important molecules for fetal development and have been shown to be expressed in the pig placenta [21]. Vascular endothelial growth factor (VEGF) is a signaling protein that stimulates angiogenesis that has an anti-apoptotic impact on endothelial cells while increasing vascular permeability and cell migration [22]. Pathways associated with hypoxia (i.e., potentially caused by reduced angiogenesis) have been implicated as important to fetal disease status during PRRSV infection [23]. These pathways were assessed in archived placental tissue from the pregnant gilt challenge model, designed to investigate the fetal host response to infection and genotypic and phenotypic factors that confer resistance and resilience [23]. The objective of this study was to assess placental targeted structural and transporter-related gene expression patterns. We hypothesize that differences in gene expression patterns will be identified between fetal groupings with varying classifications of PRRSV infection. The novelty of the current study is the investigation of the groups of fetuses with varying levels of demise (i.e., VIA, MECm, and MECs). This study was a logical next step to the previous publication [10] where gene expression in the placenta more accurately predicted fetal demise compared to that in the thymus. The investigation of structural and transporter groupings using gene expression also gives novel insights into potential mechanisms that contribute to variation in fetal demise.

## Results

### Fetal phenotypic characteristics and groups

Supplemental Fig. S1 delineates the experimental design including fetal groupings. An attempt was made to balance numbers of fetuses between each group (range = 13–21/group). However, the resulting distribution was representative of the larger population of fetuses this group was selected from. Detailed information pertaining to fetal phenotypic characteristics including VL (fetal serum, placenta, and thymus), morphometric measurements (fetal weight, crown rump length, brain/liver ratio), and their classification for resilience is presented in Table 1. The VL in the placenta, serum, and thymus were significantly (*P* < 0.05) higher in HVF compared to LVF groups (Table 1). Fetal weight was significantly (*P* < 0.05) higher in VIA_HVF compared to VIA_LVF groups. No significant differences were identified between groups for crown to rump length. The brain: liver ratio was significantly (*P* < 0.05) higher in VIA_LVF compared to MECs_HVF groups; no other differences between groups were identified.

**Table 1:** Summary of morphometrics and PRRSV viral load by fetal resilience groups

Group	Classification	*N*	Sex (male, %)	Placental viral load	Serum viral load	Thymus viral load	Fetal weight (g)	Crown rump length (cm)	Brain/ Liver ratio
CTRL	NA	14	50%	ND^A^	0 (0)^A^	ND^A^	822.64 (33.76)^AB^	27.31 (0.85)^A^	1.25 (0.05)^AB^
VIA_LVF	A combination of resilient and tolerant	15	47%	3.91 (0.46)^B^	1.50 (0.27)^B^	0 (0.00)^B^	610.77 (39.89)^B^	25.13 (0.56)^A^	1.56 (0.10)^A^
VIA_HVF	Tolerant	21	38%	7.00 (0.24)^C^	7.12 (0.19)^C^	5.23 (0.20)^C^	808.54 (58.23)^A^	27.62 (0.73)^A^	1.36 (0.09)^AB^
MECm_LVF	Susceptible	14	71%	4.58 (0.64)^B^	1.56 (0.27)^B^	0 (0.00)^B^	769.46 (63.97)^AB^	27.45 (0.86)^A^	1.25 (0.11)^AB^
MECm_HVF	Highly susceptible	14	64%	7.79 (0.29)^C^	7.33 (0.47)^C^	5.13 (0.20)^C^	754.26 (48.40)^AB^	26.88 (0.62)^A^	1.20 (0.07)^AB^
MECs_HVF	Extremely susceptible	13	62%	7.29 (0.18)^C^	8.05 (0.24)^C^	5.57 (0.28)^C^	759.22 (35.06)^AB^	26.14 (0.57)^A^	1.02 (0.16)^B^

Fetal phenotypes for groups based on PRRSV viral load as determined by quantitative PCR on fetal placenta, serum and thymus, in the form of group average in log10 copies/µL. Numbers for phenotypic measurements are displayed as the mean and standard error in parentheses. Further subdivision of fetuses was made based on preservation status at the time of sample collection with only fetuses classified as either viable (VIA), meconium stained mild (MECm), and meconium stained severe (MECs) used in the present work. Group control (CTRL) was mock infected, viable and low virus fetus (VIA_LVF), viable and high virus fetus (VIA_HVF), meconium mild and low virus fetus (MECm_LVF), meconium mild and high virus fetus (MECm_HVF), and meconium severe and high virus fetus (MECs_HVF). Classifications NA (not applicable as not challenged). The fetal sex is reported as percent male for the given category. Different letters within column indicate significant (*P* < 0.05) differences between fetal groups for the given phenotype

### Differentially expressed genes

The numbers and directions of differentially expressed genes (DEG) by contrast group are summarized in Table 2. Supplemental Table S1 contains contrast results from every gene in the form of log2FC. No DEG were identified in MECm_LVF and a single upregulated DEG was identified in VIA_LVF, each contrasted to CTRL. In the VIA_HVF group, 7 DEG were identified and 57% (*n* = 4/7) were upregulated. In the MECm_HVF group, 29 DEG were identified and 62% (*n* = 18/29) were upregulated. In the MECs_HVF group 39 DEG were identified, with 15% (*n* = 6/33) upregulated.

**Table 2:** Number and directionality of differentially expressed genes (DEG) by contrast

Contrast (group 1 – group 2)		Placenta		
Fetal group 1	Fetal group 2	Down-Regulated DEG	Up-Regulated DEG	Total DEG
VIA_LVF	CTRL	0	1	1
VIA_HVF	CTRL	3	4	7
MECm_LVF	CTRL	0	0	0
MECm_HVF	CTRL	11	18	29
MECs_HVF	CTRL	33	6	39

Each group of fetuses was contrasted with the control group. Group control (CTRL) was mock infected, viable and low virus fetus (VIA_LVF), viable and high virus fetus (VIA_HVF), meconium mild and low virus fetus (MECm_LVF), meconium mild and high virus fetus (MECm_HVF), and meconium severe and high virus fetus (MECs_HVF). Analysis for DEG was completed in Limma and those reported had a *P* ≤ 0.05

### Overlap of DEG

**Fig. 1:**
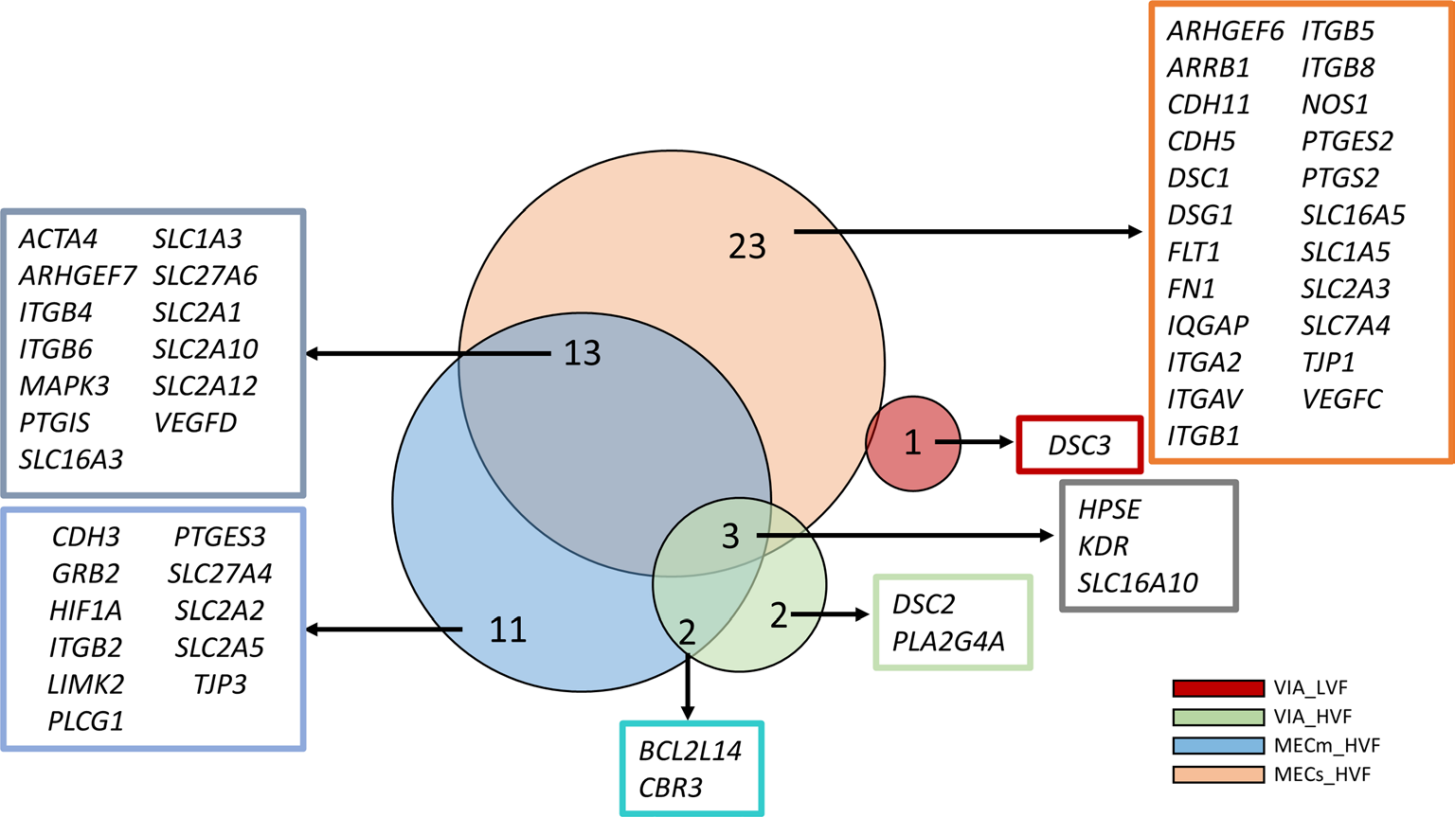
Proportional-area Venn Diagrams depict unique and shared numbers of differentially expressed genes (DEG) in fetal placenta among fetal contrast groups. Each group of fetuses was contrasted with the control group. Group control (CTRL) was mock infected, viable and low virus fetus (VIA_LVF in red), viable and high virus fetus (VIA_HVF in green), meconium mild and high virus fetus (MECm_HVF in blue), and meconium severe and high virus fetus (MECs_HVF in orange). Meconium mild and low virus fetus (MECm_LVF) had no DEG and is not included). Analysis for DEG was completed in Limma and those reported were significant at *P* ≤ 0.05. Bubble sizes are based on relative numbers of DEG for a given contrast calculated using the Euler method

Unique and shared DEG were identified and visualized using proportional area Venn Diagrams with overlap of individual groups contrasted with CTRL (Fig. 1). A core set of three DEG were identified in the highly infected (VIA_HVF, MECm_HVF, and MECs_HVF) fetal groups, which were *HPSE*,* KDR*, and *SLC16A10*, all of which were downregulated (contrasted to CTRL). A large proportion of the DEG in the MECs_HVF were unique to this group (23/39 = 60%) and had genes representative of all gene groupings investigated here. A large proportion of the MECm_HVF were shared with DEG of the MECs_HVF group (13/29 = 45%). Various individual genes unique and shared among contrasts are listed in Fig. 1.

### Fetal sample clustering based on infection severity using principal component analysis

A principal component analysis (PCA) was completed using the log2FC, from each fetal group compared to CTRL, for the 86 test genes to identify clustering (Supplemental Fig. S2A). The log2FC of genes with the highest magnitude loading values (i.e., either positively or negatively) were visualized to investigate expression patterns associated with the PCA clustering (Supplemental Fig. S2B). Supplemental Table S2 contains the loading values associated with each of the 86 test genes. A total of 57.5% of the variance was explained by Principal Component 1 (PC; PC1). PC1 contained fetuses with low VLs and/or viable fetuses loaded positively (on the right side) while fetuses with high VL and MEC staining loaded negatively (on the left side). The top four positive loaded genes on PC1 were *ARHGEF6*, *CDH11*, *IQGAP*, and *SLC7A4*. The top four negative loaded genes on PC1 were *ARHGEF7*, *ITBG4*, *ACTA4*, and *SLC16A3*. Principal Component 2 (PC2) explained 27.9% of the variance. Although there was no clear grouping of fetuses, the VIA_LVF loaded most negatively (most negative on Y-axis) while MECm_HVF loaded most positively (most positively on Y-axis). The top positive and negative loaded genes onto PC2 included *NOS3* and *SLC2A5* (data not plotted).

### Gene grouping enrichment

Our NanoString gene set (*n* = 86) was selected a priori, based on gene groupings related to structural and transporter genes, that we hypothesized to be involved in fetal demise during PRRSV infection, and that also were previously known to be expressed in the pig placenta. The investigated gene groupings along with the percent enrichment are found in Table 3. The overall percent enrichment ranged from 43% in the actin cytoskeleton signaling grouping to 88% in the transportation grouping.

**Table 3:** Gene groupings, gene targets tested within each group, number of differentially expressed genes (DEG), and gene grouping enrichment

Gene group	Gene targets within group	Total DEG	Number of genes tested	Enrichment (%)
Actin cytoskeleton signaling	***ACTA4***, *ACTG1*, ***ARHGEF6***, ***ARHGEF7***, *ARPC1A*,* ARPC2*,* CDC42*,* F2RL1*, ***IQGAP***, ***LIMK2***, *RAC2*,* ROCK1*,* ROCK2*	5	13	38%
Arachidonic acid	*CBR1*,* CBR2*, ***CBR3***, *PTGES*, ***PTGES2***, ***PTGES3***, ***PTGIS***, *PTGS1*, ***PTGS2***, *TBXAS1*, ***PLA2G4A***	6	11	55%
Integrin signaling	***FN1***, ***GRB2***, ***ITGA2***, ***ITGAV***, ***ITGB1***, ***ITGB2***, *ITGB3*, ***ITGB4***, ***ITGB5***, ***ITGB6***, *ITGB7*, ***ITGB8***, *SPP1*	10	13	77%
Intracellular junctions	*CDH1*, ***CDH11***, ***CDH3***, ***CDH5***, ***DSC1***, ***DSC2***, *DSC3*, ***DSG1***, *DSG2*,* DSG3*, ***TJP1***, *TJP2*, ***TJP3***	8	13	62%
Transporters	***SLC1A3***, ***SLC1A5***, ***SLC2A1***, ***SLC2A2***, ***SLC2A3***, ***SLC2A5***, ***SLC2A10***, ***SLC2A12***, *SLC7A1*, ***SLC7A4***, *SLC16A1*, ***SLC16A3***, ***SLC16A5***, ***SLC16A10***, ***SLC27A4***, ***SLC27A6***	14	16	88%
VEGF signaling	***ARRB1***, *ARRB2*, ***BCL2L14***, ***FLT1***, ***HIF1A***, ***HPSE***, ***KDR***, *MAPK1*, ***MAPK3***, ***NOS1***, *NOS2*,* NOS3*,* PGF*, ***PLCG1***, *PLCG2*,* PTGR1*,* VEGFA*,* VEGFB*, ***VEGFC***, ***VEGFD***	11	20	55%
House Keeping	*HMBS*,* IPO8*,* MAU2*,* RPL32*	4	0	N/A

Each group of fetuses was contrasted with the control group. Group control (CTRL) was mock infected, viable and low virus fetus (VIA_LVF), viable and high virus fetus (VIA_HVF), meconium mild and low virus fetus (MECm_LVF), meconium mild and high virus fetus (MECm_HVF), and meconium severe and high virus fetus (MECs_HVF). Analysis for DEG was completed in Limma and those reported had a *P* ≤ 0.05. Bolded genes indicated they were identified as DEG in ≥ 1 contrast compared to the CTRL group. Enrichment % was calculated as the number of DEG divided by the number of genes tested multiplied by 100

**Fig. 2:**
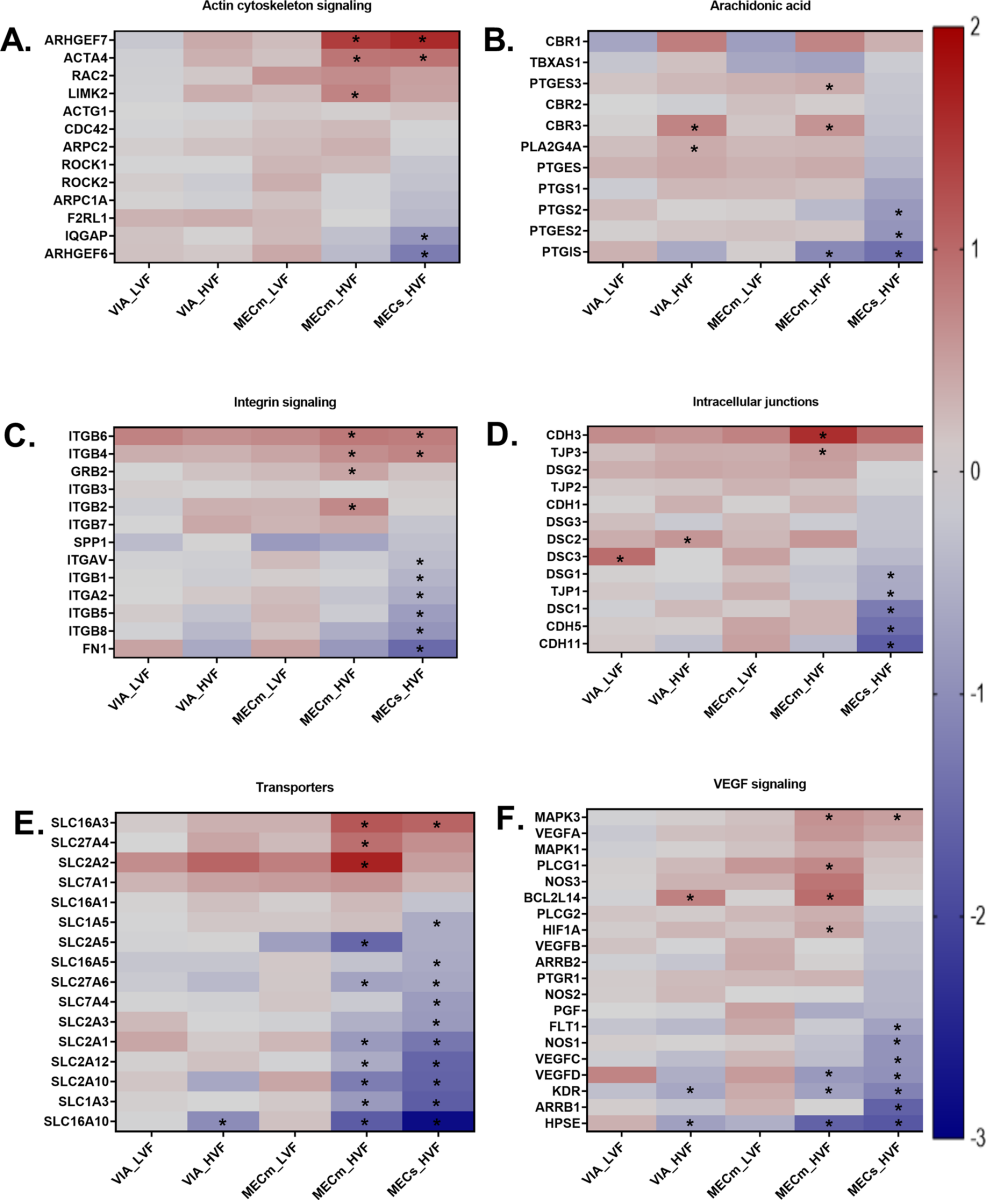
Heatmaps of log2 fold changes by gene grouping for all 86 test genes in fetal placenta for each fetal contrast group. Each group of fetuses was contrasted with the control group. Group control (CTRL) was mock infected, viable and low virus fetus (VIA_LVF), viable and high virus fetus (VIA_HVF), meconium mild and low virus fetus (MECm_LVF), meconium mild and high virus fetus (MECm_HVF), and meconium severe and high virus fetus (MECs_HVF). Each figure shows an individual gene grouping: **A**) actin cytoskeleton signaling, **B**) arachidonic acid, **C**) integrin signaling, **D**) intercellular junctions, **E**) transporters, and **F**) VEGF signaling. Note that genes within each gene grouping were sorted from high to low log2FC values in the MECs_HVF fetal group. Analyses for DEG were completed in Limma with asterisks (*) used to show significant (*P* ≤ 0.05) differences from CTRL. The color corresponds to a gradient of the log2FC values ranging from − 3 to + 2 with upregulated in red and downregulated in blue

To visualize DEG within each gene grouping, heatmaps were generated based on the log2FCs and are found in Fig. 2. The log2FC ranged from + 2 to -3 among the DEG. In general, the heatmaps showed the trend that gene expression tended to become down regulated with increasing fetal severity with MECs_HVF showing the largest proportion of downregulated genes. The gene grouping expression results are presented below as those DEG found in 1 or more contrast versus CTRL (i.e., VIA_LVF, VIA_HVF, MECm_LVF, MECm_HVF, and MECs_HVF) although the majority of DEG belonged to the MECs_HVF group. The actin cytoskeleton signaling group had 3 DEG upregulated (*ARHGEF7*,* ACTA4*, and *LIMK2*) and 2 downregulated (*IQGAP* and *ARHGEF6*). The arachidonic acid group had 3 DEG upregulated (*PTGES3*, *CBR3*, and *PLA2G4A*) and 3 downregulated (*PTGS2*, *PTGES2*, and *PTGIS*). The integrin signaling had 4 DEG upregulated (*ITGB6*, *ITGB4*, *GRB2*, and *ITGB2*) and 6 genes downregulated (*ITGAV*, *ITGB1*, *ITGA2*, *ITGB5*, *ITGB8*, and *FN1*). Intercellular junctions had 4 DEG upregulated (*CDH3*, *TJP3*, *DSC2*, and *DSC3*). Transporters had 3 DEG upregulated (*SLC2A2*, *SLC16A3*, and *SLC27A4*) and 11 downregulated (*SLC1A3*, *SLC1A5*, *SLC2A1*,* SLC2A3*,* SLC2A5*,* SLC2A10*,* SLC2A12*,* SLC7A4*,* SLC16A5*, *SLC16A10*, and *SLC27A6)*. Transporter gene grouping had the highest percent enrichment (88% or 14 of 16 genes investigated were DEG). The log2FC for these genes were plotted to better depict the gene expression patterns across fetal groupings (Supplemental Fig. S3). The VEGF signaling had 4 DEG upregulated (*MAPK3*, *PLCG1*, *BCL2L14*, and *HIF1A*) and 7 downregulated (*FLT1*, *NOS1*, *VEGFC*, *VEGFD*, *KDR*, *ARRB1*, and *HPSE*).

Across all contrasts, *SLC2A2* was the gene with the highest magnitude upregulation with a log2FC of 1.67 in the MECm_HVF vs. CTRL contrast while *SLC16A10* was the gene with the highest magnitude downregulation with a log2FC of -2.8 in the MECs_HVF vs. CTRL contrast. In the VIA_LVF vs. CTRL, the genes with the largest magnitude upregulation were *DSC3*,* ITGB6*, and *VEGFD* while those downregulated were *KDR*,* FLT1*, and *SLC16A5*. In the VIA_HVF vs. CTRL, the genes with the largest magnitude upregulation were *SLC2A2*,* BCL2L14*, and *CBR3* while those downregulated were *SLC16A10*,* HPSE*, and *KDR*. In the MECm_LVF vs. CTRL, the genes with the largest magnitude upregulation were *SLC2A2*,* CDH3*, and *ITGB6* while those downregulated were *SLC2A5*,* HPSE*, and *SLC2A3*. In the MECm_HVF vs. CTRL, the genes with the largest magnitude upregulation were *SLC2A2*,* CDH3*, and *ARHGEF7* while those downregulated were *SLC16A10*,* HPSE*, and *SLC2A5*. In the MECs_HVF vs. CTRL, the genes with the largest magnitude upregulation were *ARHGEF7*,* SLC16A3*, and *CDH3* while those downregulated were *SLC16A10*,* HPSE*, and *SLC1A3*.

## Discussion

The objective of this study was to assess placental targeted structural and transporter-related gene expression patterns. The current study resulted in four major findings: (1) fetal PRRSV infection levels (i.e., high viral load) rather than fetal demise alone (i.e., meconium staining) is necessary to alter placental gene expression; (2) placental expression of transporters from a range of solute carrier subfamilies (i.e., *SLC1A3*, *SLC1A5*, *SLC2A1*,* SLC2A3*,* SLC2A5*,* SLC2A10*,* SLC2A12*,* SLC7A4*,* SLC16A5*, *SLC16A10*,* SLC27A6*,* SLC2A2*,* SLC16A3*, and *SLC27A4)* was strongly decreased by fetal PRRSV infection; (3) placental structural gene expression (i.e., downregulation of *ARHGF6*,* FN1*, and *CDH11*; and upregulation of *ARHGEF7*,* ITGB6*, and *CDH3*) was moderately impacted by fetal PRRSV infection; and (4) expression of genes associated with arachidonic acid and VEGF signaling (i.e., downregulation of *PTGIS* and *HPSE;* and upregulation of *PTGES3* and *MAPK3*) were moderately dysregulated by fetal PRRSV infection. The understanding of the mechanisms of fetal resistance/tolerance and susceptibility to PRRSV infection are advanced by the gene expression results in this study.

### Rational for fetal selection, tissue selection, structural and transporter gene grouping selection, and experimental methods

In the current studies, we classified fetuses in the VIA_LVF group as a combination of resistant and tolerant as they remain uncompromised in the face of viral infection and they have minimized infection, suggesting the ability to prevent viral entry/replication [24]. These fetuses may also have been infected later than their siblings. We classified the MECm_LVF group as susceptible because they are compromised even at low VL [24]. The VIA_HVF are tolerant as they remain uncompromised despite high VL [25]. The MECm_HVF are highly susceptible while we consider the MECs_HVF extremely susceptible due to high VL and fetal demise. In the current study, our fetal classifications (Table 1) were based on PRRS VL and fetal meconium staining. However, our data indicate that the most resilient/tolerant group (VIA_LVF) showed signs of IUGR while our most susceptible group (MECs_HVF) showed limited evidence of the IUGR phenotype, which is consistent with previous work and was thoroughly discussed elsewhere [26]. In addition, previous work from our group identified a positive genetic correlation between VL and fetal body weight (0.44 ± 0.41), and a negative genetic correlation between VL and brain: liver ratios (− 0.97 ± 0.78) [27], indicating that genetic selection for decreased VL would result in lower fetal body weight and higher brain: liver ratio, and ultimately increased frequency of IUGR. Careful consideration would need to be given to make breeding decisions based alone on our classifications of fetal resilience because of this association with IUGR. We attempted to balance fetal sex within group since previous reports show that fetal sex can impact placental gene expression [28]. However, a very small number of DEG were identified in both groups at the ends of the range with VIA_HVF and MECm_LVF having 7 and 0 DEG, respectively. Therefore, we anticipate that fetal sex had a minimal impact on the interpretation of the results in our study. This unique dataset al.lowed comparisons across varying categories of fetal demise status. Meconium staining can be considered a systemic response to the physiological stress of infection while we are focused on implications at the site of the placenta tissue alone. Here we use meconium staining in PRRSV infected fetuses as an indication of impending fetal death. Susceptible fetuses can die in utero, be autolyzed, or born weak then die shortly after birth [7], resulting in large economic impacts to the industry. Our study investigated fetal groupings differentiated in part by the severity of fetal meconium staining in PRRSV infected fetuses. Although the non-infected fetuses in our dataset were all viable, the larger challenge experiment did contain a small proportion of meconium stained fetuses that were in the CTRL group (7/97 = 7%). However, PRRSV infected fetuses more than doubled the occurrence of meconium staining in our larger challenge experiment (65/395 = 16%), indicating a strong association between infection and meconium staining. Nevertheless, meconium staining has been reported to be relatively common [29], indicating that some of the variation in meconium staining identified within our study may be due to natural variation within the pig population.

The genes selected were hub genes (i.e., genes with various interactions) and genes known or hypothesized to be involved in response to PRRSV infection for each gene grouping. Compared to RNAseq technology, NanoString is a more sensitive measure of gene expression and less dependent on RNA quality [30, 31]. However, NanoString analyses is dependent on the CodeSet used and may therefore miss genes that are not expected to be associated with host response to PRRSV infection. A limitation of our study is that it is a simplification of a complex system. We utilized one breed/line of gilts, one PRRSV strain, and no way of knowing when individual fetuses were infected so the duration of infection cannot be controlled (i.e., MEC may be infected longer than VIA). Our study focused on a limited number of genes (86) to investigate specific hypotheses (i.e., structural and transported related gene expression associations with PRRSV infection severity) using one gene expression assay methodology (NanoString). Additional verification could be done to compare our results to other expression assay methods, as well as additional assays. For example, previous studies in the pregnant gilt model have gained valuable insight from investigating protein abundance [32], cell type composition [33], and histological assays [12] among others to provide additional insight into the mechanisms of PRRS resistance.

We selected genes within six transporter and structural gene groupings, each are discussed below. The actin cytoskeleton and the associated signaling transduction pathways are involved in regulation of cellular shape, movement, and pathogen cell invasion among other functions [17]. Previous research on congenital Zika virus infection reported changes in the extracellular integrity of the placenta via actin remodeling through IFN-beta, indicating this may be an important factor in disruption to the placental integrity and resulting in fetal infection [34]. Changes in gene expression in actin cytoskeleton signaling may impact the ability of the placenta to structurally prevent or enable PRRSV transmission to fetuses. The mechanism of fetal infection is still not completely understood.

Arachidonic acid is an essential fatty acid, a major component of cell membranes, and is a substrate for the synthesis of prostaglandins and other eicosanoid hormones which collectively have diverse endocrine functions [18]. Previous work (Van Goor et al., 2019) has shown genes within the arachidonic acid pathway were upregulated in more severely PRRSV infected fetuses compared to uninfected. These genes may contribute to the proinflammatory response. When their expression is reduced, disease severity is reduced due to limiting immune over responsiveness [35].

Integrins are transmembrane proteins that mediate cellular adhesion [19] and have a critical role in mammalian pregnancy [36], including in the pig placenta [37]. Previous research has reported an association of decreased expression levels of integrins with reduced growth in fetuses [38]. Because of the association of intrauterine growth restriction (IUGR) with improved fetal outcome in response to PRRSV infection [26], these integrins may be particularly important. Thus, integrin expression could provide insights into placental adhesion changes in response to PRRSV infection.

Intracellular adhesion molecules, including tight junctions are for pericellular complexes that control permeability [20]. Previous studies from our group reported some changes in tight junction protein expression among fetuses with varying degrees of disease progression in the placenta of PRRSV infected fetuses, however, there was no evidence of widespread down regulation of intercellular junction proteins in compromised fetuses [39].

Transportation proteins are critical to the primary function of the placenta, which is to provide vital resources to the developing conceptus. These transporters are divided into four main superfamilies; ATP-binding cassettes, ATPases, ion channels, and SLCs [40, 41]. Here, we selected a subset of the large solute carrier family that function in the transportation of glucose, amino acids, and fatty acids because they are among the most important molecules supporting fetal development and have been shown to be expressed in the pig placenta [21].

VEGF is a signaling protein that stimulates angiogenesis, has an anti-apoptotic impact on endothelial cells, and increases vascular permeability and cell migration [22]. Pathways associated with hypoxia (i.e., potentially caused by reduced angiogenesis) has been implicated as being important to fetal outcome during PRRSV infection [23]. Inhibition of angiogenesis in pig placenta has been shown to be associated with reduced growth in fetuses [42]. All 6 pathways we investigated were enriched (i.e., 38–88% of genes tested were DEG or enriched in each group) in one or more contrasts in this study, providing evidence for the value of the selected set of genes and their associated pathways.

### Impact of fetal viral load on structural and transporter gene expression

Our data support the concept that fetal PRRSV viral levels (i.e., high VL), rather than fetal demise alone (i.e., meconium staining), are necessary to alter structural and transporter gene expression levels in the placenta. We detected only one DEG, desmocollin-3 (*DSC3)*, in VIA_LVF fetuses and did not detect any DEG in the MECm_LVF group, indicating the regulation of structural and transporter gene expression, at least at 12 DPMI, is not responsible for generating the combination of resistant and tolerant (VIA_LVF) fetal phenotype. DSC3 is a cell adhesion protein, which increased in gene expression in the current study. *DSC3* has been reported to have expression across a wide range of tissues, with the highest expression in the ovary [43]. In addition, the overall gene expression patterns assessed by PCA revealed a strong grouping mainly by fetal VL (Supplemental Fig. S2). A key finding of our study was the lack of gene expression response of the placenta when fetal VL is low indicates that perhaps the structural and transporter genes have a more important functional role in fetal demise than vertical transmission, which was a similar finding for immune related gene expression from our group [10]. Although our study did not identify genes and their expression patterns that are associated with tolerance, besides some evidence for *DSC3*, we do report genes associated with susceptibility.

Our results show that once fetuses are classified as HVL, a strong change in gene expression among structural and transporter genes is initiated within the MFI. Upon high VL infection, a set of core responsive DEG found in the transporters and VEGF signaling gene groupings (*KDR*, *HPSE*, and *SLC16A10*) were significantly (*P* < 0.05) downregulated in the fetal placenta. Additional details on the impact of each gene grouping are discussed below in order of transporters, structures, arachidonic acid, and VEGF signaling.

### Importance of transporters on fetal demise

A key finding of our study is that our data suggests the dysregulation of transporter genes is involved in fetal demise. Sixteen SLC transporter genes were tested that function in transportation of glucose, amino acids, and fatty acids across the MFI to the developing fetus. Overall, 88% (14/16) in this group were identified as DEG in ≥ 1 contrast, which made it the most enriched pathway investigated in the current study. The DEG identified in MECm_HVF and/or MECs_HVF versus CTRL contrasts suggests the dysregulation of transporters (i.e., downregulation of *SLC1A3*, *SLC1A5*, *SLC2A1*, *SLC2A3*, *SLC2A5*, *SLC2A10*, *SLC2A12*, *SLC7A4*, *SLC16A5*, *SLC16A10*, and *SLC27A6*; and upregulation of *SLC2A2*, *SLC16A3*, and *SLC27A4*) may be important. In general, we detected an overall suppression of SLC transporters in highly susceptible (MECm_HVF) and extremely susceptible (MECs_HVF) fetuses compared to CTRL. Interestingly, two facultative glucose transporters (*SLC2A2* and *SLC2A5*) along with a fatty acid transporter (*SLC27A4)*, were found to be DEG only in the MECm_HVF group but not in the MECs_HVF group, indicating their potential importance in severity of the fetal response. Our data indicates an upregulation of *SLC2A2* and a downregulation of *SLC2A5* may be associated with a partially protective effect and a less severe fetal outcome. SLC2A2 enables glucose movement across cell membranes while SLC2A5 enables fructose movement. Although fructose is important in fetal development, glucose is the primary energy source [44]; the prioritization of glucose transportation over fructose transportation may provide biological insight into disease resilience/tolerance. Our CodeSet included two other major glucose transporters, SLC2A1 (GLUT1) and SLC2A3 (GLUT3). GLUT1 and GLUT3 are widespread, and transport glucose in most tissues [45]. In our current study, *SLC2A1* and *SLC2A3* were both significantly downregulated compared to CTRL in both MECm_HVF and MECs_HVF groups. These results may indicate less glucose is being transported across the placenta in these fetal groups. Previous research from our group identified SLC candidate genes (*SLC16A4*, *SLC18A3*, *SLC25A20*, *SLC26A6*, and *SLC39A7*) near a QTL for thyroid hormone levels in PRRSV infected fetuses and piglets [27]. The most well-studied solute carriers that act as thyroid hormone transporters include *SLC16A2*, *SLC16A10*, and *SLCO1C1* [46, 47], and many other transporters have been shown to interact with thyroid hormones to various degrees as reviewed previously [48]. Two previous studies from our group investigated *SLC16A10* expression in placenta of PRRSV infected fetuses and found significant downregulation in infected fetuses versus uninfected fetuses [10, 49]. Our finding is consistent as we found *SLC16A10* to have the strongest downregulation of all genes tested (log2FC of -2.8 in MECs_HVF vs. CTRL) and was DEG in all contrasts with HVF in the current study. In the current study, we carefully dissected the placental tissue from endometrium and did not measure or weigh the placental tissue. Previous research has reported a positive phenotypic correlation between fetal size and placental size [50]. Although our study normalized for tissue input using housekeeping genes, it would be reasonable to expect the total transporter protein abundance would be greater in heavier fetuses. In summary, fetal infection appears to be altering the flux of nutrients through the MFI in part via changes in transporter genes. Nutrient availability in the fetus may impact the ability of the PRRSV to replicate in the fetus. Alternatively, changes in the fetal nutrients may be the result of altered metabolic activity associated with the fetal or maternal response to infection.

### Dysregulation of structural-related gene groupings

The structural gene groupings that were investigated include actin cytoskeleton signaling, integrin signaling, and intercellular junctions. The actin cytoskeleton signaling data revealed a strong downregulation of *ARHGEF6* and a strong upregulation of *ARHGEF7* in extremely susceptible fetuses (MECs_HVF vs. CTRL). These two genes were also the top positively loaded and negatively loaded genes in the PCA score plot, supporting their importance in assessing fetal infection severity (Supplemental Fig. S2), and these taken together are key findings of our study. *ARHGEF6* and *ARHGEF7* are homologous proteins that form a complex together, are located in the cytoplasm, respond to extracellular stimuli by coupling with G proteins to stimulate Rho-dependent signals, and are negative regulators of the focal adhesion assembly [51]. Interestingly, it has been shown that expression levels of *ARHGEF7* and *ARHGEF6* are negatively correlated [52], which could explain the decrease in *ARHGEF6* and increase in *ARHGEF7* identified here. Actin stabilization is presumed to be partially activated in susceptible fetuses by the upregulation of *ACTA4* and *LIMK2* [53]. However, there was downregulation of *IQGAP*, which is an actin binding protein activator of actin polymerization, and is involved in downstream formation of adheres junctions [54]. This could indicate that actin stabilization was increased to counteract the decrease in actin polymerization. The upregulation of *ARHGEF7* accompanied by the downregulation of *IQGAP* may be involved in fetal demise by suppressing the formation of placental focal adhesions and adherens junction, which could reduce the integrity of the tissue and contribute to fetal demise.

The integrin signaling genes were dysregulated only in fetuses that were highly susceptible (MECm_HVF vs. CTRL) and extremely susceptible (MECs_HVF vs. CTRL). *ITGB6*, *ITGB4*, *GRB2*, and *ITGB2* were all upregulated in one or more of these fetal groups. The ITGs play a central role in focal adhesions, development of the actin cytoskeleton among other functions [55]. Integrins are of particular interest in non-invasive placentas, such as the pigs, where they play a role in attachment of maternal and fetal tissues. Previous work has shown an association with the expression of integrins at the MFI with fetal size; smaller sized fetuses had decreased expression of *ITGB6* [38]. Previous work from our group has shown that fetal intrauterine growth is associated with a more susceptible phenotype to PRRSV infection [26]. Alternatively, non-IUGR fetuses could exhibit infection sooner than IUGR fetuses. The *ITGB6* functions as a receptor to *FN1*, which was strongly downregulated in our study. The increased *ITGB6* expression in susceptible fetuses could be a marker for fetal size/fetal demise. *ITGB4* encodes a subunit of the integrin protein, *ITGB2* is a surface protein also known as *CD18* that functions in leukocyte adhesion [56], and *GRB2* promotes ERK/MAPK-mediated transcription with the Integrin signaling pathway [57]. These genes were all downregulated only in the extremely susceptible group (MECs_HVF) compared to CTRL. These included both alpha and beta integrins [58]. Fibronectin or *FN1* was the most strongly downregulated gene in the integrin signaling group. *FN1* is an extracellular matrix component that binds integrin receptors to influence cell adhesion and migration. The downregulation of *FN1* in highly susceptible fetuses confirmed previous work from our group [10]. This strong downregulation of *FN1* may reduce integrin signaling despite some counteractive increases in integrin binding proteins.

Intracellular junction gene expression was moderately impacted in our study (62% enrichment). *CDH3*, *TJP3*, *DSC2*, and *DSC3* were significantly upregulated in 1 or more fetal grouping. These genes are part of the class of adherens junctions, intercellular junctions, and desmosomes [59]. Interestingly, the *DSC2* and *DSC3* genes were positively DEG in both tolerant fetal groupings (VIA_LVF and VIA_HVF each vs. CTRL). The desmocollin proteins are parts of the desmosome structure that function to hold adjacent cells tightly together. From our understanding, this is the first report of the association of increased expression of *DSC2* and *DSC3* with potential increased fetal resilience. These DSCs may contribute to maintaining placental structural integrity, fetal protection and growth, resulting in fetal viability. *CDH3* is a cadherin protein involved in cellular adhesion and *TJP3* is a tight junction protein that forms linkages with cellular actin cytoskeleton and tight junctions; both were upregulated only in the MECm_HVF compared to CTRL. This could indicate one mechanism of distinction between mild versus severe meconium staining. *CDH11* and *CDH5* were most strongly downregulated in the MECs_HVF group (compared to CTRL), giving insight into the potential association of cadherin disruption in the placenta with extreme fetal susceptibility.

### Importance of arachidonic acid and VEGF signaling on fetal demise

The arachidonic acid genes were moderately impacted in this study. We found upregulation of *PTGES3*, *CBR3*, and *PLA2GA4*, in one or more groups compared to CTRL. *PTGES3*, Prostaglandin E synthase 3, is an enzyme involved in the proper functioning of glucocorticoid and other steroid receptors. There is a negative correlation between glucocorticoid levels and pro-inflammatory cytokines [60], which could be partially associated with the mild meconium staining. *CBR3* is a reductase that functions in the conversion of prostaglandin E to F2a and *PLA2GA4* cytosolic phospholipase A2. This study identified a down regulation of *PTGS2*, *TBXAS1*, and *PTGIS* in the meconium stained group vs. viable group. *PTGS2* functions in prostaglandin E synthesis and PTGIS is required for prostacyclin production and have been implicated in response to PRRSV infection [61]. Overall, there appeared to be a downregulation of arachidonic acid signaling.

The VEGF signaling genes were moderately impacted in our study (55% enrichment). Four genes were upregulated and seven were downregulated. The VEGF signaling pathway is involved in stimulating angiogenesis, which may be increased in response to hypoxic fetal conditions. Previous work has indicated that decreased angiogenesis at the MFI is associated with fetal compromise, except in cases of IUGR where inherently lower angiogenesis may have a protective effect [62]. We hypothesized that VEGF signaling gene expression may be increased in resilient fetal groupings, which could contribute to increased angiogenesis in the placenta. Previous research has shown that dead PRRSV infected fetuses have a higher likelihood of umbilical cord lesions, which has been hypothesized to cause fetal death via hypoxia from reduced blood flow to the fetus [63, 64]. Previous research from our group found some signs of hypoxia in the fetal heart [65]. The results from our study indicate that PRRSV infection, particularly in the most susceptible fetuses, results in a decreased angiogenesis. *MAPK3* was most strongly upregulated in susceptible fetuses (MECm_HVF and MECs_HVF each compared to CTRL). This gene is part of the mitogen-activated protein kinases gene family that regulates diverse cellular activities [66], is a suppresser of cell growth and protein synthesis [67], and is reported to promote angiogenesis [68]. *PLCG1*, *BCL2L14*, and *HIF1A* were also upregulated in various fetal groups indicating these fetuses may have been experiencing hypoxia, which has previously been reported by our group [65]. It has been shown that PRRSV infection in the fetus results in changes in cellular proliferation and decreased angiogenesis at the MFI [62]. Hypoxia generally increases HIF1A gene expression, which promotes angiogenesis [69]. However, previous work from our group has reported fetal compromise may be related to reduced uterine submucosal angiogenesis, except for IUGR fetuses where inherently reduced angiogenesis may be associated with a protective effect against PRRSV infection [62]. However, the majority of genes in this grouping were downregulated; with *HPSE* and *ARRB1* showing the most significantly reduced log2FC compared to CTRL. Heparinase (*HPSE*) is involved in remodeling of the extracellular matrix and has been shown to release angiogenic factors, which promote tumor formation [70]. The arrestin protein *ARRB1* has been shown to interact directly with *VEGFR3* that could promote angiogenesis [71]. A downregulation in *VEGFD*, *VEGFC*, and *NOS1*, which are key components in the VEGF signaling pathway, suggests that angiogenesis may be inhibited in PRRSV infected fetuses indicating the dysregulation of this pathway may be contributing to fetal demise.

Our study provides evidence that structural and transporter gene expression changes are associated with fetal demise. However, the role these gene groupings play in vertical transmission is unclear. Our data show that there were a limited number of DEG in VIA and LVF groupings, indicating that high VL with or without fetal demise is required to initiate gene expression changes, rather than viral infection alone. Future research probing the molecular mechanisms of vertical transmission could focus on other structural protein groups such as those involved in macrophage migration across the placenta. In addition, vertical transmission could also involve free virus or clusters by existing nutrient pathways or exosome/macrovesicles, which could be a future area of research as well.

## Conclusions

We aimed to characterize mechanisms associated with variation in fetal response to PRRSV infection. We found that the fetal structural and transporter pathways in the placenta had many changes in gene expression in response to PRRSV infection, indicating the importance of these genes in fetal demise. The transporter genes, particularly those that transport glucose, amino acids, and fatty acids, were largely downregulated as fetal severity increased, which may be a mechanism leading to fetal demise. This study identified that gene expression of structural genes was impacted and appeared to support the hypothesis that placenta structure is changed by PRRSV infection which may make fetal infection or survival worse. Moreover, arachidonic acid and angiogenesis appeared to be decreased in highly susceptible fetuses. Our unique dataset allowed us to investigate the molecular mechanisms that may contribute to variation in fetal demise during PRRSV infection. The results of our study can be used in the fight against PRRS in the future by providing groundwork to continue investigating structural impacts of PRRS on the MFI. The gene expression associations identified here may be utilized to improve host response to PRRSV infection through breeding or therapeutic development.

### Methods

#### Animal experiments and fetal classification

Our study aimed to investigate the molecular mechanisms of fetal disease resistance/tolerance and susceptibility to PRRSV infection. Pregnant, purebred Landrace gilts, were purchased from a high-health nucleus heard (Fast Genetics Inc, Spiritwood, SK) that were artificially inseminated with homospermic semen from one of 24 Yorkshire boars (Fast Genetics Inc, Spiritwood, SK) as described previously [72]. Authors were non-blinded to group allocation at the different stages of the experiment. In the current dataset, pregnant gilts at 84 (± 0.4) days gestation were randomly chosen to be infected (*N* = 22 of 31 gilts in this dataset) with 1 × 105 TCID_50_ PRRSV (NVSL97-7895); 2 mL intramuscular (IM) and 1 mL into each nostril or mock infected (*N* = 7 of 7 gilts in this dataset) similarly with MEM as described in detail previously [8]. Gilts were observed twice daily for clinical signs of infection and behavior. Gilt rectal temperatures and feed intake were assessed once daily following challenge. Gilts were euthanized at 12 DPMI by administering intravenously a solution of 30 mL of pentobarbital sodium (16,2000 mg) per gilt diluted with equal parts sterile water, followed by cranial captive bolt and exsanguination. This injection provides ~ 80 mg/kg, sufficient to euthanize the gilt as well as fetuses, as pentobarbital quickly crosses the placenta. The uterus was removed, and fetal preservation status determined, including the severity and location of meconium staining. The MFI samples were carefully dissected to separate placental tissue from endometrium. The VL in placenta, fetal thymus, and fetal serum were determined as described previously [7]. A summary of morphometrics (fetal weight, crown rump length, and brain to liver ratio) and PRRSV VL by fetal resilience groups is found in Table 1. Fetuses were categorized by PRRS VL as assayed in the thymus and serum (i.e., low virus fetus: LVF, high virus fetus: HVF) and preservation status (i.e., viable: VIA, meconium stained mild: MECm, and meconium stained severe: MECs) (Table 1 and Supplemental Fig. S1) into disease resistance/tolerance and susceptibility groups: CTRL, VIA_LVF, VIA_HVF, MECm_LVF, MECm_HVF, and MECs_HVF. Meconium staining severity was assessed on all fetuses. In the current study, all CTRL and VIA fetuses did not have any meconium staining. The fetuses classified as mild meconium staining (MECm) showed visible signs of a small amount of meconium restricted to the face or feet, while fetuses classified as severe meconium staining (MECs) showed visible signs of meconium on both the face and body, or the face alone but with a moderate amount of meconium. Fetuses lacking pulsations in the umbilical cord at the time of sampling were excluded from the dataset. A total of 92 placenta samples were selected and assayed for gene expression. While the number of samples per group was not balanced and ranged from 13 to 21 individuals per group (Table 1 and Supplemental Fig. S1) it was reflective of the fetal population. Note as discussed below, one fetal sample was removed from the MECs_HVF group resulting in 91 fetal samples listed in Table 1 and Supplemental Fig. S1. We attempted to balance fetal morphometric measurements between fetal groupings, such that the resulting subpopulations were representative of the larger population that our samples were taken from. We also attempted to balance fetuses by sex within group; the percent male fetuses ranged from 38% in the VIA_HVF group to 71% in the MECm_LVF group (Table 1). The brain: liver ratio of weights was calculated as an indicator of IUGR. Fetuses classified as VIA_LVF are a combination of resilient and tolerant as they remain uncompromised in the face of viral infection and they have, at least thus far, minimized infection suggesting some capacity to prevent viral entry/replication [24]. The MECm-LVF are considered susceptible as they are compromised even at low viral levels [24]. The VIA-HVF are tolerant as they remain uncompromised in the face of high levels of viral infection [25]. The MECm-HVF are highly susceptible while we consider the MECs-HVF extremely susceptible. Previous work from our group has demonstrated the association of IUGR with improved fetal outcome in response to PRRSV infection [26]. The fetuses classified as VIA_LVF showed signs of IUGR compared to other fetal groupings (i.e., had the lowest fetal weight and highest brain: liver ratio) (Table 1). The MECs-HVF did not show signs of IUGR (i.e., had moderate body weight and the lowest brain: liver ratio) (Table 1). Phenotypic measurements between fetal groupings were compared using ANOVA, Tukey correction for multiple testing was utilized, fetal group was as an effect in the model, JMP Pro 15.0.0 (SAS Institute) software was used, and *P* < 0.05 considered significant.

### Isolation of RNA and gene expression analyses

Placental tissue RNA was isolated using the RNeasy mini kit (P/N 74106, Qiagen) and then checked for quantity and quality using the Agilent 2200 TapeStation (Agilent Technologies). The NanoString nCounter array (NanoString Technologies) with custom made probes was used to assay 10 housekeeping genes and 89 test genes. The gene names along with the gene grouping assignments are found in S3. Supplemental Table S4 contains the NanoString counts for all housekeeping genes originally tested summarized as the average +/- standard error by fetal group including significant differences between fetal groups, which was the criteria used for removal of the 6 housekeeping genes. The NanoString counts for each fetus were tested by ANOVA for significant differences among fetal groups to detect expression stability. Note that each gene was assigned to a single gene grouping. A total of 6 gene groupings were targeted to characterize structural and transporter genes in the placenta, and the genes were previously shown to be expressed in the pig placenta. The NanoString manufacturer’s instructions were utilized for the nCounter Master kit to prepare samples for RNA quantification. Block design randomization of samples (i.e., chips were loaded with approximately the same number of fetal samples from each group) on the NanoString chips was used. Discrete count data for every gene and every sample is produced from the nCounter. The count data was produced using the manufacturer’s instructions on the NanoString nSolver Analysis Software (version 3.0, NanoString Technologies). Negative and positive control probes were assayed on the NanoString chips, which are used to correct for background and then perform normalization by incorporating the median expression values of the 4 housekeeping genes with stable expression (i.e., *MAU2*,* RPL32*,* HMBS* and *IPO8*) nSolver software. There were 3 fetal samples flagged for QC binding density by the nSolver software. According to NanoString analysis guidelines, we investigated the positive and negative control counts for these samples and then created a heatmap within fetal grouping to visualize if each flagged sample was an outlier. Based on these results we removed 1 fetal sample from the MECm_HVF group. We also removed three genes across all fetal samples: *AKR1C3*, *DSG4*, and *FLT4*, that were all flagged as not meeting quality control (QC) by NanoString software due to extremely low counts across all samples. In total, we retained 86 test genes for downstream analyses that were normalized by 4 housekeeping genes from 91 fetal samples. Consistent with our previously research [10] linear models in the Limma software package were used to calculate DEG based on the assumption of normality. Normalized counts for all test genes were used as input and are presented in Supplemental Tables S5, and were used as input into the Limma Zoom R package [73] in Galaxy [74] with the following gene expression model Y_norm counts_ = Group + Error.

### Analysis of differentially expressed genes (DEG)

The experimental unit was an individual tissue sample from a single fetus. Each group was contrasted with the CTRL group i.e., VIA_LVF vs. CTRL, VIA_HVF vs. CTRL, MECm_LVF vs. CTRL, MECm_HVF vs. CTRL, and MECs_HVF vs. CTRL. P value ≤ 0.05 was considered significant. No log2FC cutoff was used given the sensitivity of the NanoString platform, along with previous research from the current group that reported tight junction gene expression in the PLC that centered between − 2 and + 2 [39]. Supplemental Table S1 contains both the p value and false discovery rate adjusted p value for each contrast. The DEG reported in the manuscript are based on the p value cutoff. NanoString does not recommend universal multiple testing correction (www.nanostring.com). This is because the CodeSets are typically designed based on genes within pathways that are suspected to be impacted in the study, which violates the assumption of independence of significance levels between tests. In addition, the multiple testing corrections may be too conservative for experiments where the tolerance for false negatives is low. We report (Supplemental Table 1) multiple testing correction using Benjamini & Yekutieli [75]. The summary of DEG by fetal grouping are listed in Table 2. The genes within gene categories, whether each gene was identified as DEG in 1 or more contrast, and the percent enrichment of a gene grouping is displayed in Table 3. Enrichment % was calculated as the number of DEG divided by the number of genes tested multiplied by 100.

### Visualization of DEG

The Euler method was utilized to visualize proportional-area Venn diagrams using DEG as input and PCA was used to identify sample clustering, both analyses were completed using JMP Pro 15.0.0 (SAS Institute) [76]. The input into the PCA analysis was the log2FC values from individual groups vs. CTRL (i.e., VIA_LVF, VIA_HVF, MECm_LVF, MECm_HVF, and MECs_HVF) for all 86 test genes with default parameters. Loading plots, heat maps, and graphs were used to visualize results using GraphPad Prism software.

## Electronic supplementary material

Below is the link to the electronic supplementary material.

Supplementary Material 1

Supplementary Material 2

Supplementary Material 3

Supplementary Material 4

Supplementary Material 5

## Data Availability

All datasets used for this study are included in the supplementary materials. In addition, the raw gene expression counts were deposited in the Ag Data Commons repository, doi accession number: https://figshare.com/s/2f81f421cdf0f62c9fe9.

## Abbreviations

CTRL: Mock infected control
DEG: Differentially expressed genes
DPMI: Days post maternal infection
HVF: High viral load fetus
IFN: Interferon
IUGR: Intrauterine growth restriction
Log2FC: Log 2 fold change
LVF: Low viral load fetus
MEC: Meconium stained fetus
MECm: Mild meconium stained fetus with staining on the head only
MECm_HVF: Meconium mild and high virus fetus
MECm_LVF: Meconium mild and low virus fetus
MECs: Severe meconium stained fetus with staining on the head and body
MECs_HVF: Meconium severe and high virus fetus
MFI: Maternal fetal interface
PC: Principal component
PC1: Principal component 1
PC2: Principal component 2
PCA: Principal component analysis
PRRS: Porcine reproductive and respiratory syndrome
PRRSV: Porcine reproductive and respiratory syndrome virus
PRRSV2: Porcine reproductive and respiratory syndrome virus type 2
R: Resilient
S: Susceptible
SER: Fetal serum
SLC: Solute carrier proteins
T: Tolerant
VEGF: Vascular endothelial growth factor
VIA: Viable fetus
VIA_HVF: Viable and high virus fetus
VIA_LVF: Viable and low virus fetus
VL: Viral load
