# Supplementary figures and images for: Altered structural and transporter-related gene expression patterns in the placenta play a role in fetal demise during Porcine reproductive and respiratory syndrome virus infection
Source: BMC Genomics. 2025 Mar 21;26:279. doi: 10.1186/s12864-025-11397-0 (PMC11927291; doi:10.1186/s12864-025-11397-0)

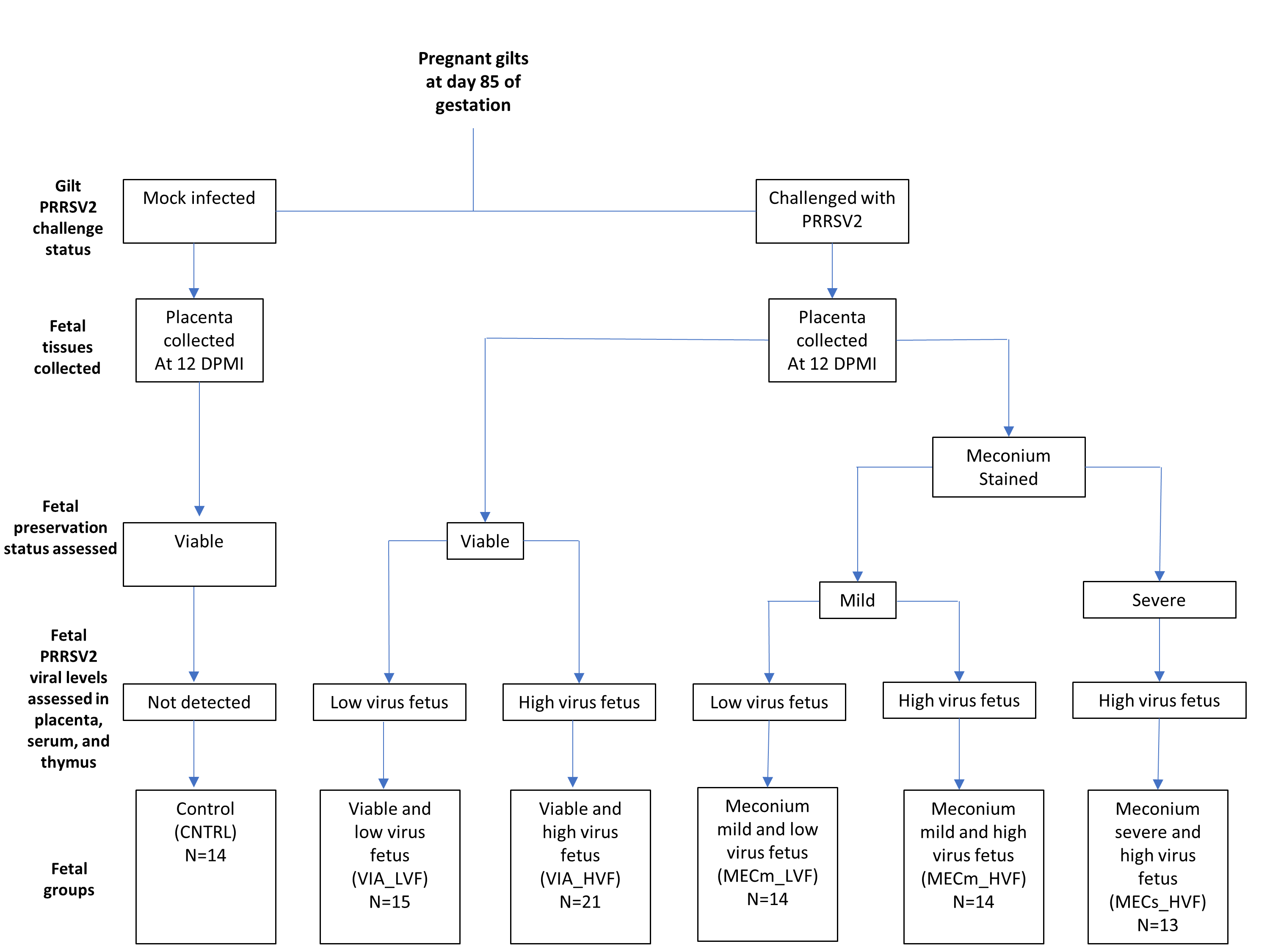

Supplement: Supplementary file 1 — Supplementary Material 1 [file 12864_2025_11397_MOESM1_ESM.tif]

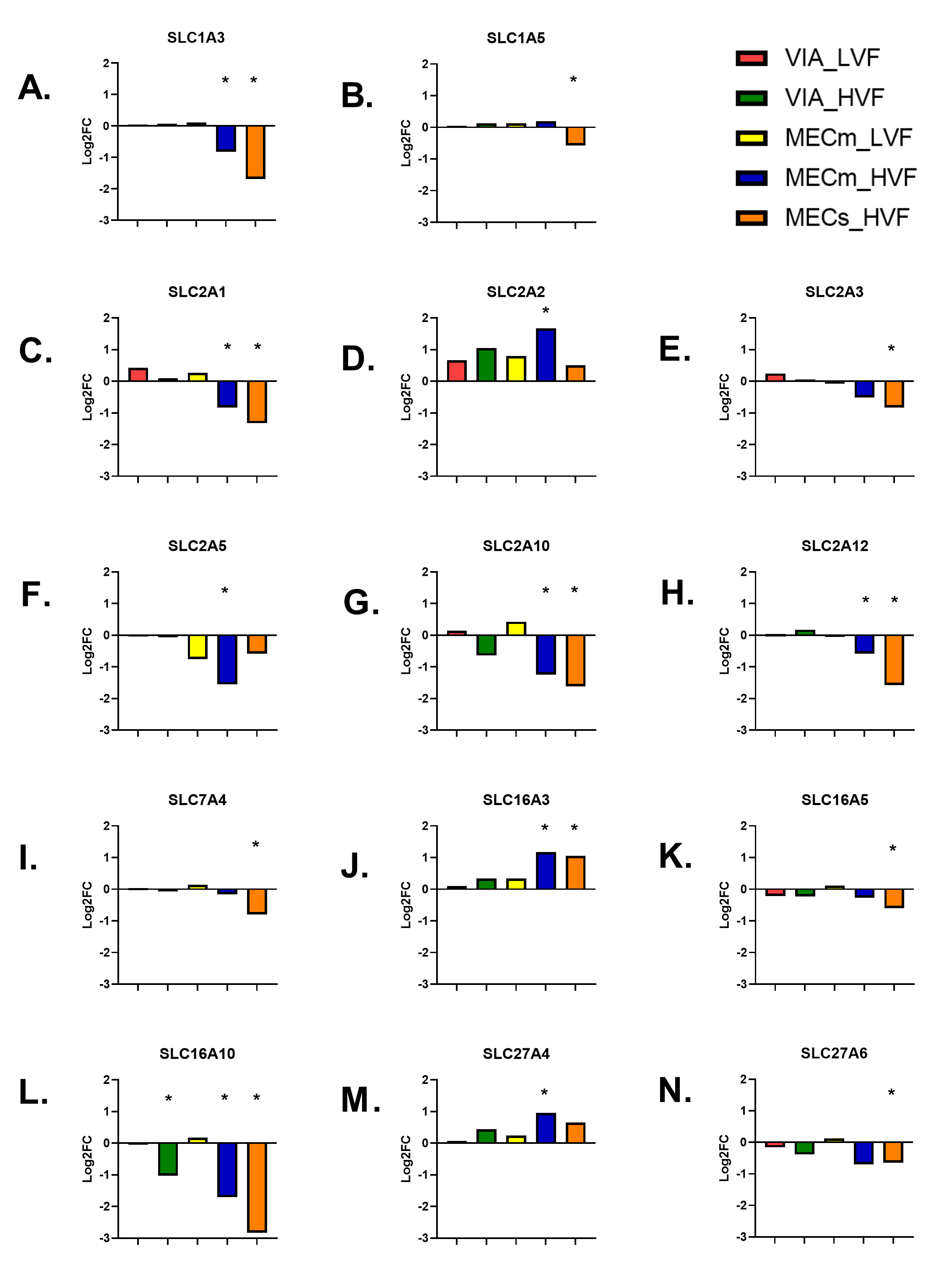

Supplement: Supplementary file 2 — Supplementary Material 2 [file 12864_2025_11397_MOESM2_ESM.tif]

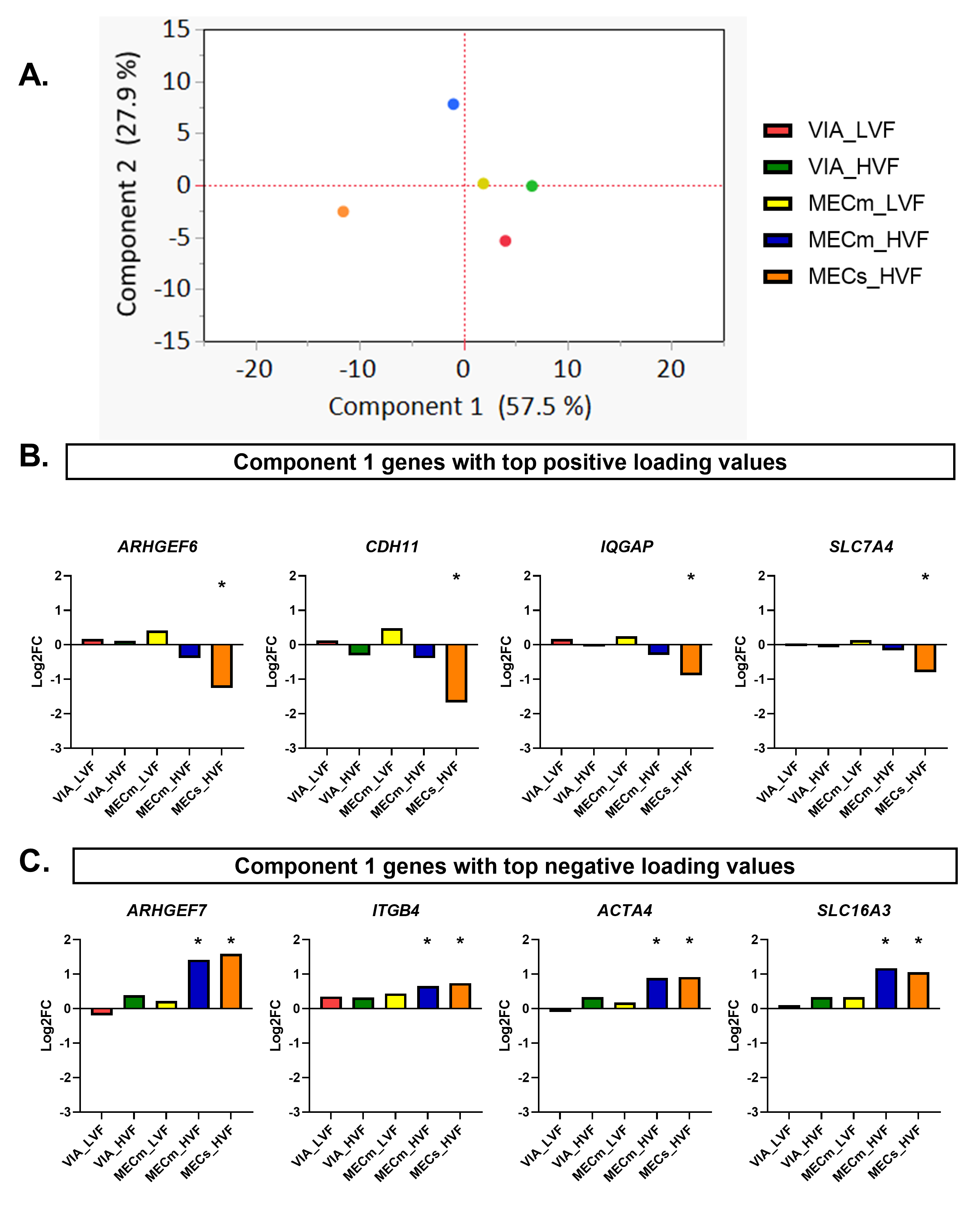

Supplement: Supplementary file 3 — Supplementary Material 3 [file 12864_2025_11397_MOESM3_ESM.tif]
