# Supplementary material for: Altered structural and transporter-related gene expression patterns in the placenta play a role in fetal demise during Porcine reproductive and respiratory syndrome virus infection
Source: BMC Genomics. 2025 Mar 21;26:279. doi: 10.1186/s12864-025-11397-0 (PMC11927291; doi:10.1186/s12864-025-11397-0)
Supplement: Supplementary file 5 — Supplementary Material 5 [file 12864_2025_11397_MOESM5_ESM.docx]

**Supplemental Information**

**Supplemental Figure 1.** Diagram illustrating how the fetal groupings were assigned. Abbreviations: porcine reproductive and respiratory syndrome virus type 2 (PRRSV2); Days Post Maternal Infection (DPMI); Group control (CTRL) was mock infected; viable and low virus fetus (VIA_LVF); viable and high virus fetus (VIA_HVF); meconium mild and low virus fetus (MECm_LVF); meconium mild and high virus fetus (MECm_HVF); and meconium severe and high virus fetus (MECs_HVF). N represents the number of fetuses that contributed placental tissue from each grouping.

**Supplemental Figure 2**. PCA score plot and genes with top positively and negatively loading values. A) Placenta PCA score plot for principal components 1 and 2. B) The top four positively loaded genes on component 1 were *ARHGEF6*, *CDH11*, *IQGAP*, and *SLC7A4.* The top four negatively loaded genes on component 1 were *ARHGEF7, ITBG4, ACTA4, and SLC16A3.* Each group of fetuses was contrasted with the control group. Group control (CTRL) was mock infected, viable and low virus fetus (VIA_LVF in red), viable and high virus fetus (VIA_HVF in green), meconium mild and low virus fetus (MECm_LVF in yellow), meconium mild and high virus fetus (MECm_HVF in blue), and meconium severe and high virus fetus (MECs_HVF in orange). The log2FC for each group contrasted to CTRL for all 86 test genes was used as input into the PCA analysis. Analysis for DEG was completed in Limma. Significant (P ≤ 0.05) group differences (within plot) are depicted with an asterisk (*).

**Supplemental Figure 3**. Transporter gene plots showing log2FC. Relative expression of 14 differentially expressed genes (DEG) found in the placenta in one or more contrast group; A) *SLC1A3*, B) *SLC1A5*, C) *SLC2A1*, D) *SLC2A2*, E) *SLC2A3*, F) *SLC2A5*, G) *SLC2A10*, H) *SLC2A12*, I) *SLC7A4*, J*) SLC16A3*, K) *SLC16A5*, L) *SLC16A10*, M) *SLC27A4*, and N) *SLC27A6*. Each group of fetuses was contrasted with the control group. Group control (CTRL) was mock infected, viable and low virus fetus (VIA_LVF in red), viable and high virus fetus (VIA_HVF in green), meconium mild and low virus fetus (MECm_LVF in yellow), meconium mild and high virus fetus (MECm_HVF in blue), and meconium severe and high virus fetus (MECs_HVF in orange). Analysis for DEG was completed in Limma. Significant (P ≤ 0.05) group differences (within plot) are depicted with an asterisk (*).
